# Hypothesis: apo‐lactoferrin–Galantamine Proteo‐alkaloid Conjugate for Alzheimer's disease Intervention

**DOI:** 10.1111/jcmm.13484

**Published:** 2018-01-29

**Authors:** Olufemi D. Akilo, Pradeep Kumar, Yahya E. Choonara, Priyamvada Pradeep, Lisa C. du Toit, Viness Pillay

**Affiliations:** ^1^ Wits Advanced Drug Delivery Platform Research Unit Department of Pharmacy and Pharmacology School of Therapeutic Sciences Faculty of Health Sciences University of the Witwatersrand Johannesburg South Africa

**Keywords:** Alzheimer's disease, apo‐lactoferrin, galantamine, proteo‐alkaloid, free radical, metal chelation, antioxidant

## Abstract

Alzheimer's disease (AD) is known to be caused by the accumulation of deformed beta amyloid and hyperphosphorylated tau proteins resulting into formation and aggregation of senile plaques and neurofibrillary tangles in the brain. Additionally, AD is associated with the accumulation of iron or metal ions in the brain which causes oxidative stress. Galantamine (Gal) is one of the therapeutic agents that has been approved for the treatment of AD, but still saddled with numerous side effects and could not address the issue of iron accumulation in the brain. The use of metal chelators to address the iron accumulation has not been successful due to toxicity and inability to address the aggregation of the plaques. We therefore hypothesize a combinatorial antioxidant–metal–chelator approach by formulating a single dosage form that has the ability to prevent the formation of free radicals, plaques and accumulation of iron in the brain. This can be achieved by conjugating Gal with apo‐lactoferrin (ApoLf), a natural compound that has high binding affinity for iron, to form an apo‐lactoferrin–galantamine proteo‐alkaloid conjugate (ApoLf–Gal) as a single dosage form for AD management. The conjugation is achieved through self‐assembly of ApoLf which results in encapsulation of Gal. ApoLf changes its conformational structure in the presence of iron; therefore, ApoLf–Gal is proposed to deliver Gal and pick up excess iron when in contact with iron. This strategy has the potential to proffer a dual neuroprotection and neurotherapeutic interventions for the management of AD.

## Introduction

AD is the most common cause of dementia, which affect mostly elderly people, and it is connected to the loss of cholinergic neurons in parts of the brain 1. AD is one of the most severe neurodegenerative disorders. Approximately 13% of people who are 65 years and above are affected by this disorder in the United States alone 2 and is the sixth leading cause of death for people age 65 years and above 3. It is projected that by 2050, there will be almost one million new cases per year. Furthermore, it is expected that there will be remarkable increase in the number of people with AD who are 85 years and above across all racial and ethnic groups. AD is characterized by loss of memory in the patient wherein the patient finds it difficult to remember things that are learnt recently coupled with failure of acquiring new information 4, 5. The symptoms noticeable at the early stage include difficulties in reasoning, effective planning, attentiveness, as well as conceptual thinking. Episodic, semantic and implicit memories are less affected than new facts and memories 6. Progressively, the patient is unable to perform most common activities of daily living as speech, reading and writing difficulties set in.

The cause of AD is associated with genetic heritability 7 as well as cholinergic 8, amyloid and tau hypotheses. However, it is widely believed that advanced age is the major risk factor of AD. The genetic factor is brought about by gene mutations on chromosome 21 in the β‐amyloid precursor protein (APP), which are responsible for early‐onset of AD in family that has history of the disease 9, 10. Cholinergic hypothesis proposed that AD is caused as a result of reduction in the synthesis of neurotransmitter acetylcholine 11. Protein misfolding disease is a characteristic of AD caused by the accumulation of deformed beta amyloid (Aβ) which resulted into senile plaques and also the accumulation of hyperphosphorylated tau proteins in the brain resulting into formation of neurofibrillary tangles. The aggregation and accumulation of these plaques and tangles in the brain have become the most prominent culprits of AD 12, 13, 14.

Iron is extremely reactive and in high concentration can lead to neuronal death 15. Iron known to be so important in the mammalian cells for metabolism and oxygen transport 16, in excess, may cause tremendous damage to the cells by way of generating free radicals and thereby causing oxidative stress which in turn triggers a destructive effects that result into AD and other neurodegenerative disorders in general 17. *Ex vivo* and *in vivo* studies have shown and established an increase in brain iron in patients with AD 15, 17, 18, 19, 20. The presence of elevated oxidative stress due to oxidation of iron has been confirmed in the brain of patients with AD 21. More recently, Raven *et al*. 15 established that increased iron resulted in tissue breakdown associated with AD. They used an MRI technique to measure the amount of brain iron in 31 patients with AD and 68 healthy subjects as control. They compared results from hippocampus and thalamus parts of the brain, and their findings suggested strongly that iron accumulation is a possible cause of AD. From the evidence of various researches, it is therefore pertinent to infer that deposition and accumulation of iron in the brain is a centre point of all the neuropathological operations which is not limited to amyloid plaques, tau phosphorylation, as well as oxidative stress in AD. There is adequate evidence to pin down interaction of Aβ and transition metal especially neocortical iron as the culprit in initiating and causing the toxicity observed in AD 22, 23, 24, 25, 26.

## Hypothesis

Several therapeutics have been researched, proposed and prepared for the treatment of AD, many of which have reached clinical trials and found to be ineffective due to some of the reasons enumerated above and are possibly discontinued because of their failure. Gal is one of the therapeutics that has a success story and approval to be used for the treatment of AD. Even then, it has not been found to be effective in treating the disease, which may be due to other factors such as accumulation of iron in the brain and free radical generation that accompanies the ailment. Combination of Gal and an iron chelator may be a promising strategy for dealing with this situation in order to obtain a more potent approach to the treatment of AD. In this study, we propose the combination of free radical generation and the metal–chelator approach to synthesize a single dosage form to manage effectively AD through ‘proteo‐alkaloid’ conjugate of ApoLf and Gal. This is proposed to serve as ‘neuroprotective–neurotherapeutic’ agent. We therefore propose Gal and ApoLf conjugate as a novel treatment for AD. This approach will act as both neuroprotection as well as neurotherapeutic interventions.

## Evaluation of the hypothesis

The successful management and treatment of AD have remained elusive despite many researches that have been carried out and those that are still ongoing. Researchers are constantly looking for effective therapy to deal with and manage this neurodegenerative disorder. Quite a number of drugs with different mechanisms of action and targets are available, and many are still under development for the management of AD. The strategies involved in making these drugs are based on firstly the amyloid hypothesis which delved into the reduction in Aβ production, its clearance and aggregate blocking 27. Due to the connection of Aβ to AD, extensive clinical trials have been carried out with drugs designed to target this peptide in a way to absolutely prevent the production of Aβ plaques. These drugs are still inefficient to prevent the cognitive decline in patients with AD 25.

Nonsteroidal anti‐inflammatory drugs such as diclofenac 28, rofecoxib 29 and indometacin 30 have been suggested to reduce the risk of developing AD; however, the efficacy of these drugs has not been proven to be effective, and in fact, all of the therapeutics developed based on amyloid‐β approaches that reached Phase III clinical trials were unsuccessful 31. The second strategy is based on cholinergic hypothesis which involves the use of cholineesterase inhibitors capable of reducing the hydrolysis of acetylcholine. Therapeutic agents such as Gal, rivastigmine and donepezil are examples of drugs developed based on the cholinergic approach (approved for the management of mild‐to‐moderate AD). The clinical profiles of these therapeutic compounds are somewhat successful. The use of these drugs for treating patients with AD improves the cognitive functions of the patients and generally gives symptomatic relief to the patients; however, the efficacy of these compounds is still very low because only about 35% of the patients respond positively to the treatments with these drugs 32. These compounds are also associated with serious side effects such as diarrhoea, vomiting and nausea 33 which place them in an inconvenient state.

Our strategy focuses on lowering brain metal ions and targeting their interaction with Aβ peptide. Metal ions mediate generation of free radical and thereby result into oxidative damage of the brain. Employing the administration of metallo‐complex therapy is a new and promising therapy for AD. Fang Du *et al*. 34 showed in their study that hepcidin considerably minimized brain iron in rats overloaded with iron. Desferrioxamine is a chelator approved by FDA for iron overload; this chelator has been known to offer some benefits in the treatment of AD. Deferiprone is another chelator that binds iron and aluminium ions, and this was approved only in Europe. The use of chelators is however limited due to their inability to effectively cross the BBB and their being neurotoxic due to lack of elimination process of the metal–chelator complex from the brain 35.

### Gal and Alzheimer's intervention

Gal hydrobromide is an alkaloid obtained from various plants such as Galanthus caucasicus, Galanthus woronowii, Narcissus and Lycoris species. It is a neuroactive therapeutic agent approved by both the European Medicines Agency (EMA) and USA Food and Drug Administration (FDA). Its primary function is to inhibit the activities of the enzyme called acetylcholinesterase; this enzyme hydrolyses the neurotransmitter acetylcholine and then followed by the cessation of synaptic transmission. The use of Gal prevents this reaction and thereby allows the survival of acetylcholine for normal neurotransmission function in patients with AD. Recently, Traykova *et al*. 36 in their *in vitro* experiment found out that Gal compound is a scavenger of reactive oxygen species.

Additionally, Gal may also form a complex with nicotinic acetylcholine receptors by binding onto it, and thereby improving phagocytosis of microglial amyloid‐beta peptides 37. However, its clinical function is limited by its low bioavailability in the brain due to the blood–brain barrier. Furthermore, it is associated with numerous side effects when administered systemically 38. Own to its ability of scavenging free radicals, Gal may help to treat oxidative stress due to free radicals generated within the brain of patients with AD and hence may act as a neurotherapeutic agent.

### ApoLf and metal chelation

ApoLf is a glycoprotein that has a strong binding affinity for iron. This cationic iron‐binding globular glycoprotein is a member of the transferrin family. It has a molecular mass of about 80 kD and consists of 892 amino acids 39, 40. It was first discovered in human milk 41, and it is also known to exist in body fluids such as saliva, tears, vaginal fluids, semen, bile, gastrointestinal fluids, urine, nasal and bronchial secretions 42. ApoLf is made up of two homologous domains which are called N‐ and C‐lobes; the two globular lobes are folded and connected by a short α‐helix. N‐lobe is made up of N1 and N2 subdomain; likewise, C‐lobe is made up of C1 and C2 subdomain with one iron site and one glycosylation site at each lobe 43. The N‐lobe consists of amino acid residues 1–333, while the C‐lobe consists of amino acid residues 345–692 44.

The main function of ApoLf is transportation of iron to the cells as well as regulation of free iron in the blood and external secretions. ApoLf also serves as a vital component of the biological functions including immunoregulatory role 45, anti‐inflammatory 46, antioxidant 47 and most importantly the exertion of its antimicrobial functions against viral, fungal and bacterial pathogens 48. ApoLf is also known to have anticarcinogenic function 49, and it is a targeting molecule for improving brain delivery of therapeutic agents 50. The iron‐binding capability is what we are focusing on in this hypothesis as this can be exploited in the clearance of excess iron in the brain of patients with AD 51.

With respect to the role of ApoLf in neurodegenerative conditions caused by hypoxia, ApoLf is known to act as a normoxic mimetic of hypoxia and is capable of stabilizing hypoxia‐inducible factor‐1 alpha (HIF‐1α) and HIF‐2α 52, 53. These findings are very important as such bioactivity can provide neuroprotection against the toxicity of prion protein 54. Guo and coworkers, 2017, reported that in addition to activation of HIF‐1α, ApoLf regulated the ERK1/2‐CREB signalling pathways and inhibited the cognitive decline in a murine model of AD 55.

### ApoLf–Gal

The current treatments for AD aside from not being very effective and their association with various side effects, they only give symptomatic relief of the disease and not total cure of the dementia. Therefore, combination of two of these strategies in form of a single dosage form may however be a promising and more effective way of treating AD. Lf has been conjugated with various compounds in other to increase its functionality. Nojima *et al*. 56 conjugated Lf with branched polyethylene glycol using N‐hydroxysuccinimide coupling method to improve its half‐life. Additionally, Lf has been conjugated to pegylated nanoparticles to form Lf nanoparticles employing thiolation reaction 57, 58. Furthermore, superparamagnetic iron oxide nanoparticles (SPION) have been conjugated to Lf using NHS/EDC coupling chemistry to form Lf‐SPION nanoparticles 59.

**Figure 1:**
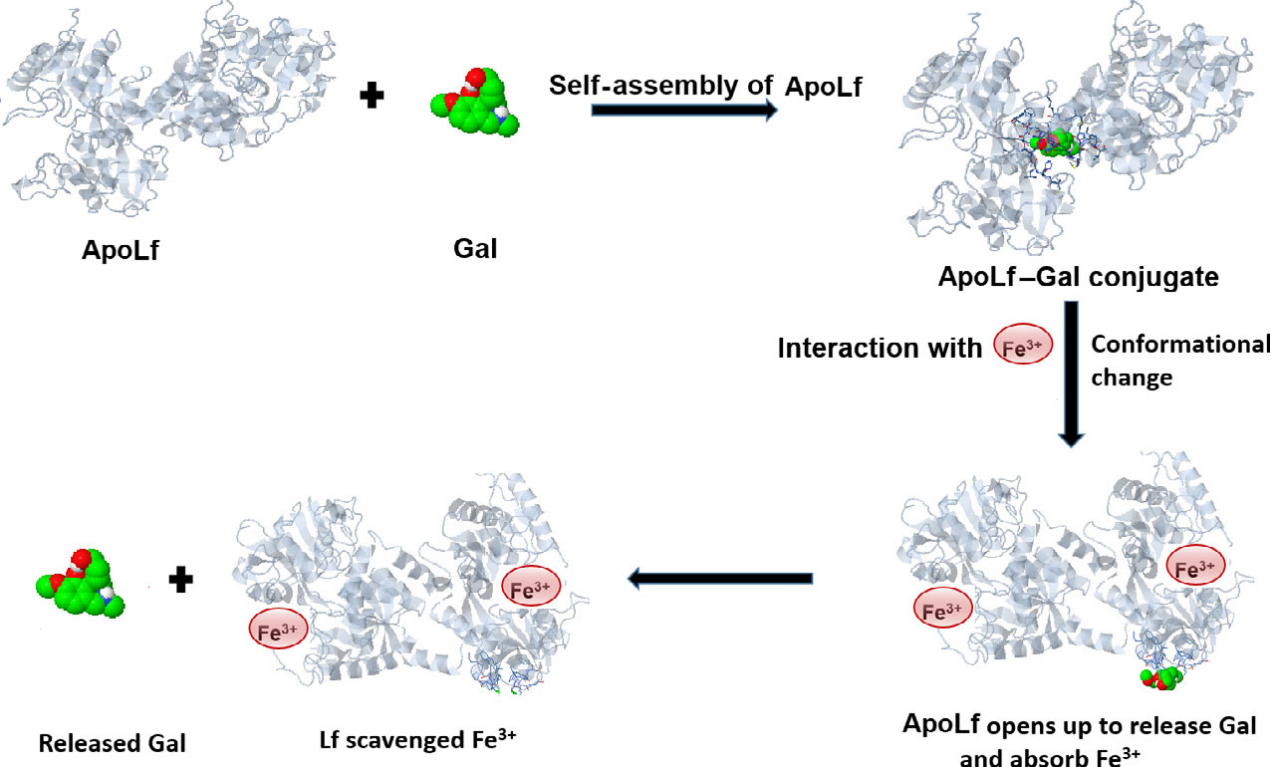
Schematic diagram of the synthesis, mechanism of release of galantamine and absorption of iron by ApoLf–Gal conjugate.

In this study, we propose to conjugate ApoLf to Gal by ‘proteo‐alkaloid’ conjugation method through the self‐assembly functionality of ApoLf. It is proposed that ApoLf may encapsulate the Gal molecules within its physical structure holding the Gal molecules inside its cavity while the ApoLf molecules self‐assemble to form a protective shell around the Gal molecule. Hydrogen bonding as well as van der Waal forces is predicted to contribute to stability of the complex formation. The demonstration of this concept is illustrated in Figure 1. This technique may be applied in developing a dosage form. Lf in itself is known to be ligand for brain targeting 50, 60, and this attribute will therefore aid the penetration of the therapeutic agent into the brain. When the conjugate is delivered into the AD patient's brain, the release of the Gal will only happen when there is interaction of the conjugate with Fe3^+^ wherein the conjugate will open up and release the Gal molecules encapsulated within the ApoLf molecule and at the same time Fe3^+^ are attracted to the ApoLf and be absorbed into it.

**Figure 2:**
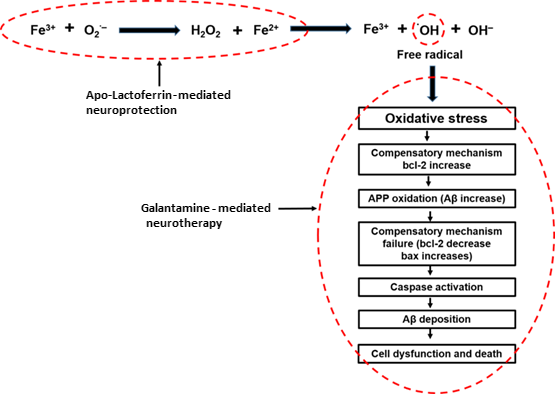
Schematic diagram demonstrating the potential neurorescue (neuroprotective–co‐neurotherapeutic) interventional mechanism of the ApoLf –galantamine conjugate.

The mechanism of conjugation and release is shown in Figure 1 where the conjugate was prepared by self‐assembly of ApoLf and upon sensing accumulation of Fe3^+^ liberates the encapsulated Gal. The release can be explained by the fact that Lf changes conformational structure whenever it interacts with Fe^3+^. Lf deprived of Fe^3+^ is known as ApoLf, while those that are saturated with iron are known as holo‐lactoferrin. The transition from ApoLf to holo‐lactoferrin which happens when ApoLf is in contact with Fe3^+^ results into conformational change accompanied with protease resistance 61. Therefore, when the conjugate is in Fe^3+^ environment, the conjugate will change its structural conformation and thereby cause the encapsulation to open up. It is envisaged that this process will allow Gal to be released, while Fe^3+^ is been absorbed into the ApoLf as it has a very strong affinity for Fe^3+^. The implication of this process will result into Gal being released to stop the activities of the free radicals produced through iron‐initiated oxidative stress and the ApoLf mops up the excess iron in the brain of the patient with AD. This is a dual action intervention that offers both neurotherapeutic and neuroprotective advantage. ApoLf due to its ability to capture excess iron in the brain may help in mediating the production of free radical that might result into the generation of oxidative stress which may eventually lead into the death of neuronal cells. Furthermore, any free radical generated will be scavenged by the action of the Gal. The process of the generation of free radical by iron in the brain, the outcome of oxidative stress and the point at which ApoLf–Gal can mediate the process is shown in the schematic diagram in Figure 2. This hypothesized technique may be a promising future strategy for managing AD.

To ascertain the hypothetical binding of Gal and ApoLf to form the proteo‐alkaloid complex, quantitative molecular docking studies were performed and the degree of ligand–protein interaction was determined, as detailed elsewhere by the authors 62. In such molecular docking studies, the final binding energy of the ligand–protein complex indicates the affinity of the ligand to the protein molecule with negative energy an indicative of preferable binding.

**Figure 3:**
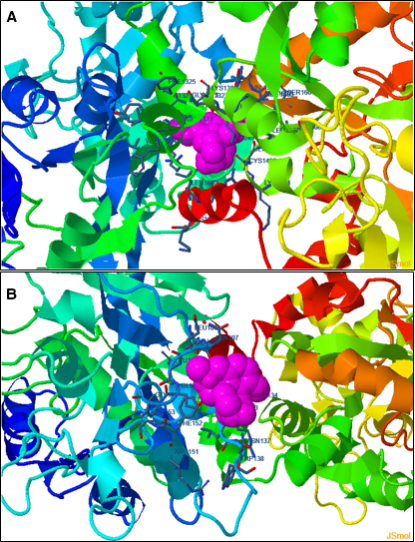
Preliminary *in silico* molecular interaction analysis of galantamine with apo‐lactoferrin (PDB ID: 1CB6) and iron‐saturated lactoferrin (PDB ID: 1LFG). The most favourable pose of galantamine (pink molecule in sphere rendering) within (**A**) the apo‐lactoferrin casing (rainbow structure in ribbon rendering); and (**B**) the iron‐saturated lactoferrin casing (rainbow structure in ribbon rendering). The docking calculations were carried out using DockingServer with the aid of AutoDock tools.

**Figure 4:**
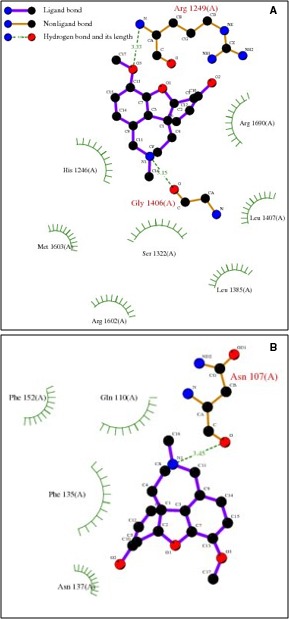
Molecular docking data depicting the 2D plot showcasing the interacting side chains and bond lengths within (**A**) ApoLf/galantamine proteo‐alkaloid complex; and (**B**) iron‐saturated Lf/galantamine proteo‐alkaloid complex. The docking calculations were carried out using DockingServer with the aid of AutoDock tools.

In this study, the ApoLf/Gal complex demonstrated a negative established free energy of binding (−6.50 kcal/mol) resulting from various intermolecular energies such as van der Waals forces, hydrogen bonding and desolvation energy along with and a large interaction surface with a frequency of 100% occurrence. The bond lengths were >3Å which confirmed the formation of a loosely bound ligand–protein complex (tightly bound complexes having bond lengths in the range of <2Å) (Figs 3 and 4). Further molecular docking simulations were performed to ascertain the affinity of Gal towards iron‐saturated lactoferrin (Lf; diferric lactoferrin). The Lf/Gal complex showed a higher free energy of binding (−4.88 kcal/mol) as compared to the ApoLf/Gal complex presenting decreased affinity of Gal towards iron‐saturated lactoferrin. Importantly, the established inhibition constant for ApoLf/Gal complex (17.33 μm) was 15 times less than that of Lf/Gal complex (263.09 μm) further confirming the reduced affinity of Gal towards iron‐saturated Lf. Interestingly, the Gal molecule in ApoLf/Gal complex was positioned in the N‐ and C‐lobe overlapping region, while in case of iron‐saturated Lf/Gal, the Gal molecule was positioned almost outside the protein molecule. The above observations can be deduced into two hypothetical assessments: (*i*) that the Gal may have high affinity towards ApoLf forming a deliverable proteo‐alkaloid complex; and (*ii*) the formation of a loose complex and less affinity of Gal towards iron‐saturated Lf may assist in the release of Gal as soon as ApoLf interacts with Fe^3+^ ions *in vivo*.

## Conclusions and implications of the hypothesis

This hypothesis demonstrated the theoretical analysis of the conjugation of Gal, an effective anti‐cholinergic agent, and ApoLf, a protein with strong binding affinity for iron. The combination of these two compounds as one dosage form with their iron and free radical scavenging ability, wherein, ApoLf with the ability to mop up excess iron in the brain and Gal acting as an antioxidant. This hypothesis may come to life by going further in carrying out the experiment in the laboratory to determine the potential of the conjugation as well as the *in vitro*,* ex vivo* and *in vivo* stability testing of the proteo‐alkaloid so developed. It may however be suggested that the conjugate be embedded in a polymer matrix or be attached to nanoparticles that possess the ability to serve as a vehicle to propagate and consequently deliver the conjugate into the brain *via* a direct nose‐to‐brain pathway. This approach may present a formidable dual neuroprotection and neurotherapeutic interventional approach and may lead to be a novel advancement in the treatment of AD.

## Conflict of interests

The authors confirm that there is no conflict of interests.
